# NMDA receptor antagonists attenuate intrathecal morphine-induced pruritus through ERK phosphorylation

**DOI:** 10.1186/s13041-018-0379-2

**Published:** 2018-06-28

**Authors:** Le Shen, Weijia Wang, Siyu Li, Jing Qin, Yuguang Huang

**Affiliations:** 1 Department of Anesthesiology, Peking Union Medical College Hospital, Beijing, 100730 China; 2 Department of Anesthesiology, The first hospital of Jilin University, Changchun, Jilin, 130021 China

**Keywords:** Intrathecal morphine, pruritus, analgesia, NMDA receptor antagonist, ERK

## Abstract

Pruritus is the most common complication of intrathecal morphine; however, its exact molecular mechanism is unclear, and treatment is challenging. The analgesic effect of N-methyl-D-aspartate (NMDA) receptor antagonists and the morphine-associated increase in NMDA receptor activation suggest potential role of NMDA receptor in the spinal itch sensation. Male C57BL/6 mice were given intrathecal morphine to induce scratching behavior. The effects of NMDA, ketamine, ifenprodil and U0126 on morphine-induced pruritus and analgesia were evaluated also. The number of scratching responses was counted for 30 min post-injection to evaluate pruritus. A warm-water tail immersion assay was conducted before and until 120 min post-injection at 30-min intervals. Percent of maximal possible effect (%MPE) and area under curve (AUC) were calculated based on tail-flick latency to evaluate analgesic efficacy. Compared with control treatment, intrathecal morphine elicited an obvious scratching response and analgesic effect in a dose dependent manner. Ketamine (1 μg), ifenprodil (0.1 μg) and U0126 (0.1 μg and 1.0 μg) all significantly attenuated morphine induced scratches. Ifenprodil (0.1 μg) injection significantly prolonged the analgesic effect of intrathecal morphine. The ERK1/2 phosphorylation induced by intrathecal morphine was inhibited by ketamine, ifenprodil and U0126 as well. U0126 inhibited morphine-induced pruritus with no effect on its analgesia. Therefore, intrathecal coadministration of morphine with NMDA receptor antagonists ketamine and ifenprodil alleviated morphine-induced scratching. Intrathecal morphine increased ERK phosphorylation in the lumbar spinal dorsal horn, which may be related with morphine-induced pruritus, and was counteracted by NMDA receptor antagonists.

## Introduction

Pruritus is the most common complication of intrathecal morphine analgesia. Various kinds of medications with different mechanisms of action have been used for the prevention and treatment of morphine-induced pruritus. However, the effects are variable and have failed to lead to the identification of a consistent mechanism of action [1, 2].

Neurons expressing gastrin-releasing peptide receptor (GRPR) have been suggested to be responsible for itch sensation in the spinal cord. The μ-opioid receptor (MOR) isoform, MOR1D, which is heterodimerized with GRPR in the spinal cord, relays itch information induced by intrathecal morphine [3, 4]. Meanwhile, another isoform, MOR1, is required for the analgesic effect of morphine [4].

Currently, the most effective treatment of intrathecal morphine-induced pruritus is an MOR antagonist, such as naloxone. Since these antagonists are unable to discriminate between MOR1 and MOR1D, the analgesic effect of morphine might be impaired [2, 5]. Recently, glutamate has been found to participate in the process of itch sensation at the level of the spinal cord through activating GRP-sensitive spinal neurons [6, 7]. Moser et al. found that morphine application to the central nervous system was capable of inducing hyperknesis [8], a process much like hyperalgesia. Considering the important role of activated glutamate receptors, especially the N-methyl-D-aspartate receptor (NMDAR) and its NR2B subunit, in central sensitization and the pathogenesis of neuropathic pain [9], the NMDAR may also be involved in morphine-induced pruritus.

NMDARs are widely distributed in the central nervous system. NMDAR antagonists, such as ketamine, have long been used as effective analgesics for either acute or chronic pain [10, 11], potentially enhancing the analgesic effect of intrathecal morphine. Furthermore, the NR2B selective antagonist ifenprodil, which acts mainly at the level of the spinal cord, might be more specific for hyperalgesia and have fewer side effects than less selective NMDAR antagonists [12].

Since extracellular signal-regulated kinase (ERK) activation is required for itch sensation in the spinal cord, in this study, we also explored whether ERK1/2 activation is related to morphine-induced pruritus at the molecular level. We used an intrathecal morphine-induced analgesia and pruritus model in mice to investigate the therapeutic effects of NMDAR antagonists, including ketamine and ifenprodil, on intrathecal morphine-induced pruritus, as well as the effects on morphine-induced analgesia. We hypothesized that ERK1/2 phosphorylation is related to intrathecal morphine-induced pruritus and that NMDAR antagonists prevent the activation of itch neurons in the spinal cord through the phosphorylation of ERK1/2.

## Methods

### Animals

The study was approved by the Ethics Committee of Peking Union Medical College Hospital (PUMCH), China. Male C57BL/6 mice (15-20 g), bred at the Institute of Laboratory Animal Science, Chinese Academy of Medical Sciences, were used in the present study. The animals were housed under laboratory conditions with controlled temperature (24°C±2°C) and free access to food and water.

### Drugs and reagents

Morphine (10 mg/ml, Yichang Humanwell Pharmaceutical Co., Ltd. China) and ketamine (50 mg/ml, Fujian Gutian Pharmaceutical Co., Ltd. China) were diluted in sterile saline. NMDA (Selleck, S7072), ifenprodil (Sigma-Aldrich, I2892) and the ERK1/2 phosphorylation inhibitor U0126 (Selleck, S1102) were dissolved in 10% DMSO. The drugs were administered intrathecally at a volume of 5 μl. p44/42 Erk1/2 Mouse mAb and phospho-p44/42 Erk1/2 Mouse mAb were obtained from Cell Signaling Technology (Beijing, China).

### Scratching behavior

Before experiments, mice were given 1 h to acclimate to a plastic chamber (15×15×20 cm), which was also used as an observation chamber after injection. Mice were removed from the chamber to receive intrathecal injection of morphine, NMDA, ketamine, ifenprodil, morphine + ketamine, morphine + ifenprodil or morphine + U0126 at different doses. The intrathecal injection method in conscious mice was described by Carolyn A. Fairbanks [13]. Briefly, the experimenter held the mouse firmly and gently by the pelvic girdle. A 30-gauge, 10-μl Hamilton syringe was inserted at the midline between the hip bones, and subarachnoid puncture was indicated by a reflexive lateral flick of the tail. Following a 5-μl injection, the syringe was rotated and removed. The mouse was then returned to the plastic chamber. One scratching response was defined as the hind limb lifting towards the back and neck of the body and then returning to the floor regardless of the number of scratching strokes that occurred between these two movements. Scratching behavior was video recorded immediately after the injection for 30 min, and the number of scratching responses were quantified. The antagonists were given intrathecally together with morphine.

### Tail immersion assay

A warm-water tail immersion assay was conducted to evaluate the analgesic efficacy [4]. The tails of the mice were half-way immersed in 52°C water warmed in a water bath. The latency to withdrawal was measured before the injection (pre-drug latency) and after the injection (post-drug latency) at 30-min intervals until 120 min after injection, with a cut-off time of 10 s. The latency was measured 3 times for each mouse at each time point, and the average was taken. Percentage of maximum possible effect (%MPE=[post-drug latency – pre-drug latency]×100/[cut-off time – pre-drug latency]) was used as an evaluation of analgesia.

### Western blotting

Tissue samples were collected from lumbar dorsal horns of the spinal cord at 5 min after injection and stored at -80°C. The tissues were homogenized and sonicated in RIPA (Conway Biotech, China). After centrifugation at 12000 g, the supernatants were collected to determine protein concentration by BCA kit (Solarbio, China). Then, equal amounts of protein were separated on SDS PAGE Bis-Tris 5-10% gels (Solarbil, China) and transferred to polyvinylidene fluoride membranes (GE Healthcare). The blots were blocked with blocking buffer (5% nonfat dry milk in TBST and 0.1% Tween-20) for 1 h at room temperature and then incubated with rabbit-anti p44/42 Erk1/2 mAb (1:10,000), rabbit-anti phospho-p44/42 Erk1/2 mAb (1:10,000) and rabbit-anti glyceraldehyde 3-phosphate dehydrogenase (GAPDH; 1:10,000) overnight at 4°C, followed by incubation in goat-horseradish peroxidase-linked secondary antibodies (1:2000) for 1 h at room temperature. Immunoblots were developed with enhanced chemiluminescence reagents (Conway Biotech, China), and the intensities of protein bands were quantified by the ImageQuant LAS4000 Mini detection system (GE Healthcare, Sweden).

### Statistical analysis

All data were expressed as the mean±standard deviation (SD), and error bars represent standard error of the mean (SEM). Statistical comparisons were performed with one-way analysis of variance (ANOVA) followed by Newman-Keuls Multiple Comparison Test. *p*<0.05 was considered statistically significant.

## Results

### Morphine-induced pruritus

**Fig. 1:**
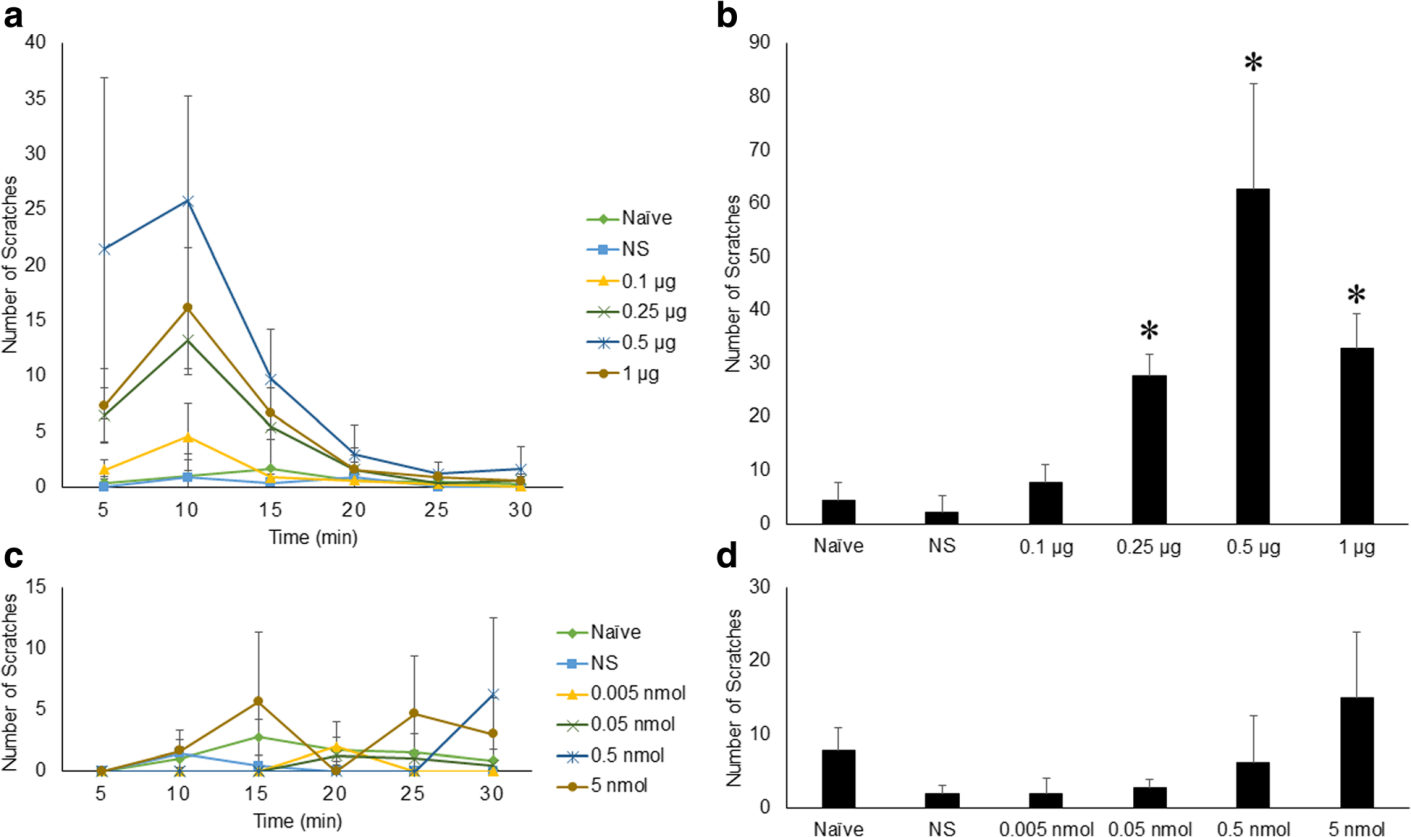
Intrathecal morphine-induced pruritus in mice. **a** Time course of the effect of morphine on scratching behavior over 30 min. Intrathecal administration of 0.5 μg morphine induced an obvious scratching response in mice, with the peak presenting in the first 5 min post-injection. **b** Dose effect of morphine on the total number of scratches in 30 min. Intrathecal administration of 0.5 μg morphine caused significant pruritus in mice compared with the other treatments. **c** & **d** Time course of the effects of intrathecal NMDA on scratching behavior over 30 min. NMDA from 0.005 nmol to 5 nmol could not induce scratching responses in mice at any time point or in 30 min. Error bars represent SEM. *: *p*<0.05, compared to the NS group

Mice were randomly assigned to the naïve group, which received no injection, the normal saline (NS) group, which received an intrathecal injection of NS, and intrathecal morphine groups, which received morphine injections from 0.1 μg to 1.0 μg. As shown in Fig. 1, the morphine-induced scratching response increased in a dose-dependent manner when the morphine dose increased from 0.1 μg to 0.5 μg. The scratching response did not further increase when 1.0 μg morphine was administered. Compared with the other groups, the mice given 0.5 μg intrathecal morphine exhibited significant pruritus. Time-course analysis revealed that the peak scratching response occurred at 5 min after the injection of 0.5 μg morphine and then quickly decreased, almost completely subsiding by 15 min after injection. Importantly, NMDAR activation through intrathecal injection of NMDA from 0.005 nmol to 0.5 nmol had no effect on the scratching response (Fig. 1).

### Morphine-induced analgesia

**Fig. 2:**
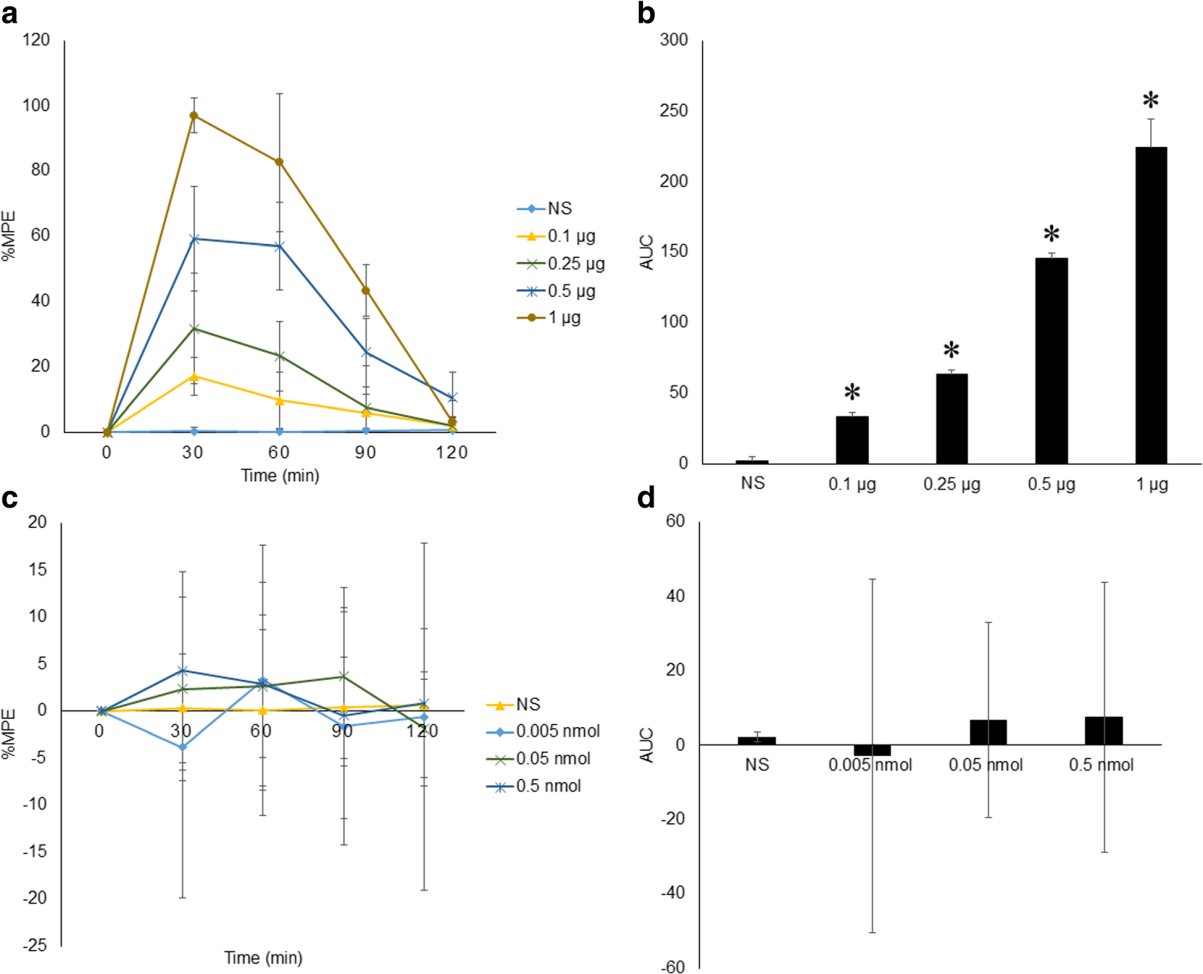
Intrathecal morphine-induced analgesia in mice. **a** Time course of the effects of intrathecal morphine on morphine-induced analgesia over 2 h post-injection. %MPE was calculated based on tail-flick latency. The %MPE was 0 pre-injection, and the peak was at 30 min post-injection, which is different from the timing of the peak of morphine-induced pruritus. **b** Dose effect of morphine on AUC calculated based on %MPE. AUC represents the overall analgesic efficacy and increased in a dose-dependent manner. **c** & **d** Time course of effects of intrathecal NMDA on %MPE and AUC calculated based on %MPE showed no analgesic effect of NMDA with the dosage ranging from 0.005 nmol to 5 nmol. Error bars represent SEM. *: *p*<0.05, compared to the NS group

In contrast to the scratching response, morphine-induced analgesia, measured by the tail immersion test and shown as %MPE, peaked at 30 min after injection. The tail-flick latency returned to baseline 2 h post-injection. In addition, the area under curve (AUC) was further calculated based on the time course of %MPE, showing that the analgesic efficacy of intrathecal morphine increased in a dose-dependent manner when the morphine dose increased from 0.1 μg to 1.0 μg. As shown in Fig. 1, 0.5 μg morphine induced obvious scratching behavior and a significant analgesic effect in mice; therefore, 0.5 μg was the dose used in the following experiments. Meanwhile, intrathecal injection of NMDA from 0.005 nmol to 0.5 nmol showed no analgesic effect (Fig. 2).

### NMDAR activation potentiated morphine-induced pruritus and decreased its analgesia

**Fig. 3:**
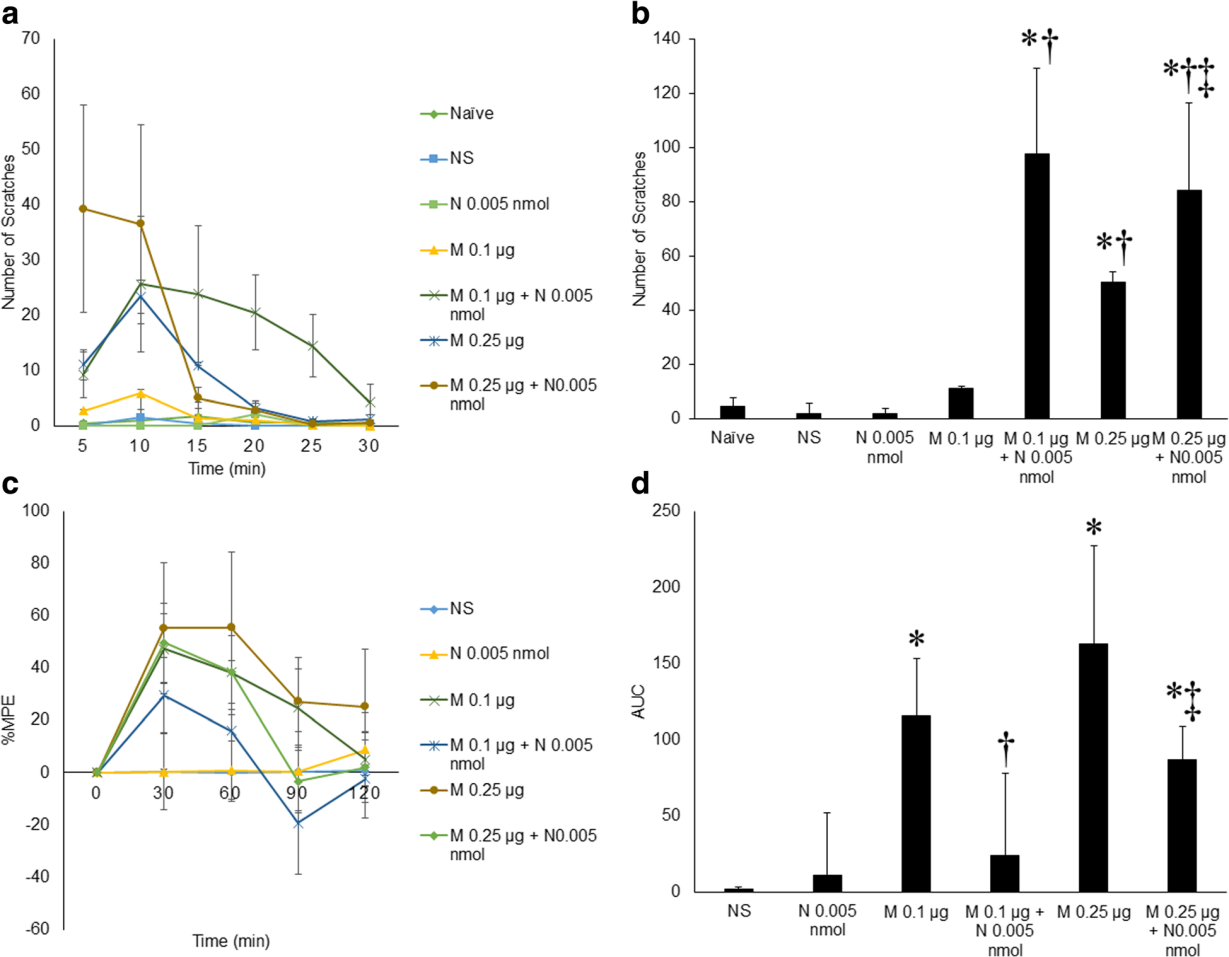
NMDAR activation potentiated morphine-induced pruritus and decreased its analgesia. **a** Morphine-induced pruritus (M 0.1 μg or M 0.25 μg) was significantly increased by 0.005 nmol NMDA. **b** NMDA (0.005 nmol) significantly increased the total number of scratches in 30 min that was induced by 0.5 μg morphine. **c** Time course of the effects of intrathecal morphine and intrathecal NMDA on morphine-induced analgesia over 2 h post-injection. NMDA injection significantly reduced the time course of analgesia induced by intrathecal morphine at either 0.1 μg or 0.25 μg. **d** The effect of ketamine and ifenprodil on AUC calculated based on %MPE in (**c**). Compared with the AUCs in the morphine groups (M 0.1 μg or M 0.25 μg), the AUCs in the morphine + NMDA groups were both significantly reduced. Error bars represent SEM. *: *p*<0.05, compared to the NS group. †: *p*<0.05, compared to the 0.1 μg morphine group. ‡: *p*<0.05, compared to the 0.25 μg morphine group

NMDA (0.005 nmol) was mixed with either 0.1 μg or 0.25 μg morphine and injected intrathecally. Compared with the scratching response in the groups given 0.1 μg or 0.25 μg intrathecal morphine, the scratching response 30 min post-injection was dramatically enhanced in the groups given 0.1 μg morphine + 0.005 nmol NMDA or 0.25 μg morphine + 0.005 nmol NMDA, respectively (total number of scratching responses: 11.00±1.06 vs. 97.60±31.57 and 50.33±3.87 vs. 84.25±32.30, respectively, *n*=9, p<0.05). When NMDAR was activated by 0.005 nmol NMDA, intrathecal morphine-induced analgesia was attenuated by 80% and 50% in the 0.1 μg morphine + 0.005 nmol NMDA group and in the 0.25 μg morphine + 0.005 nmol NMDA group, respectively (%MPE: 115.55±37.59 vs. 23.77±53.99 and 163.34±64.02 vs. 86.99±21.87) (Fig. 3).

### Ketamine and ifenprodil attenuated morphine-induced pruritus and potentiated its analgesia

**Fig. 4:**
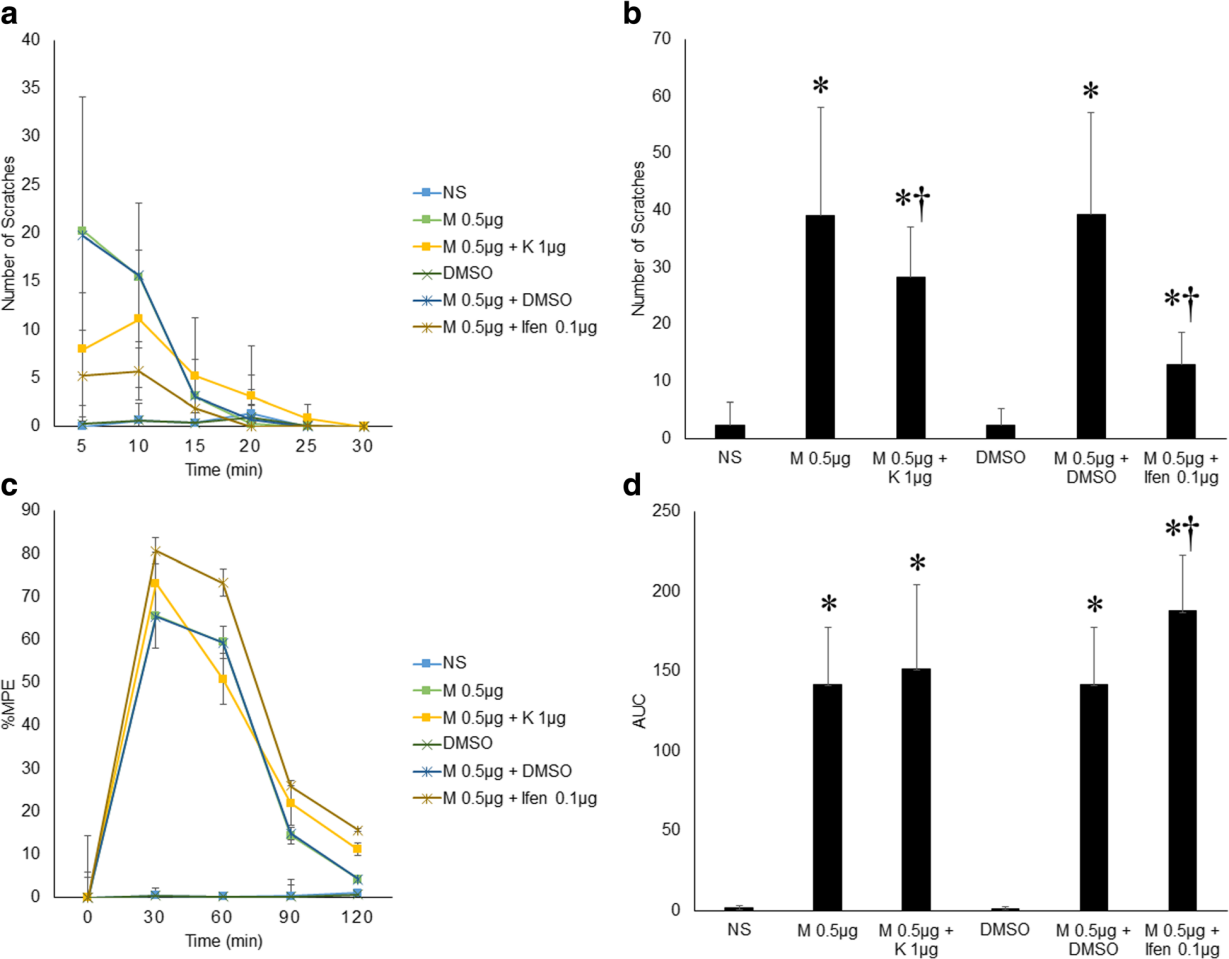
Ketamine and ifenprodil attenuated morphine-induced pruritus and potentiated its analgesia. **a** Morphine-induced pruritus (M 0.5 μg or M 0.5 μg + DMSO) was obviously impaired by 1.0 μg ketamine or 0.1 μg ifenprodil. **b** Ketamine (1.0 μg) and ifenprodil (0.1 μg) significantly decreased the total number of scratches in 30 min that was induced by 0.5 μg morphine. **c** Time course of intrathecal morphine, intrathecal morphine + ketamine, and morphine + ifenprodil on morphine-induced analgesia over 2 h post-injection. Ketamine did significantly affect the time course of analgesia induced by intrathecal morphine. %MPE was elevated by ifenprodil at 30, 60, 90 and 120 min post-injection. **d** The effect of ketamine and ifenprodil on the AUC calculated based on %MPE in (**c**). Compared with the AUCs in the control groups (NS or DMSO), the AUCs in the morphine group, morphine + 1.0 μg ketamine group and morphine + 0.1 μg ifenprodil group were all significantly increased. Compared with the AUC in the 0.5 μg morphine group, the AUC in the 0.5 μg morphine + 0.1 μg ifenprodil group was significantly increased. Error bars represent SEM. *: *p*<0.05, compared to the NS or DMSO group. †: *p*<0.05, compared to the 0.5 μg morphine group or 0.5 μg morphine + DMSO group

Most importantly, this study examined the effect of NMDAR antagonists, ketamine and ifenprodil, on morphine-induced pruritus and analgesia. In this study, 1 μg ketamine and 0.1 μg ifenprodil were tested. Compared with the scratching response in the intrathecal 0.5 μg morphine group, the scratching response 30 min post-injection was significantly attenuated in the 0.5 μg morphine + 1 μg ketamine and 0.5 μg morphine + 0.1 μg ifenprodil groups (total number of scratching responses: 39.13±18.98 vs. 28.38±8.63 and 39.25±17.99 vs. 13.00±5.61, respectively, *n*=9, *p*<0.05). Ketamine injection (1.0 μg) slightly but nonsignificantly changed the time course of %MPE in mice given 0.5 μg morphine (141.60±36.10 vs. 151.60±52.69, *n*=9, *p*>0.05). However, 0.1 μg ifenprodil injection significantly prolonged the time course of %MPE in the mice given 0.5 μg morphine (141.70±35.73 vs. 187.60±34.99, *n*=9, *p*<0.05) (Fig. 4).

### Ketamine and ifenprodil decreased ERK1/2 activation associated with intrathecal morphine

**Fig. 5:**
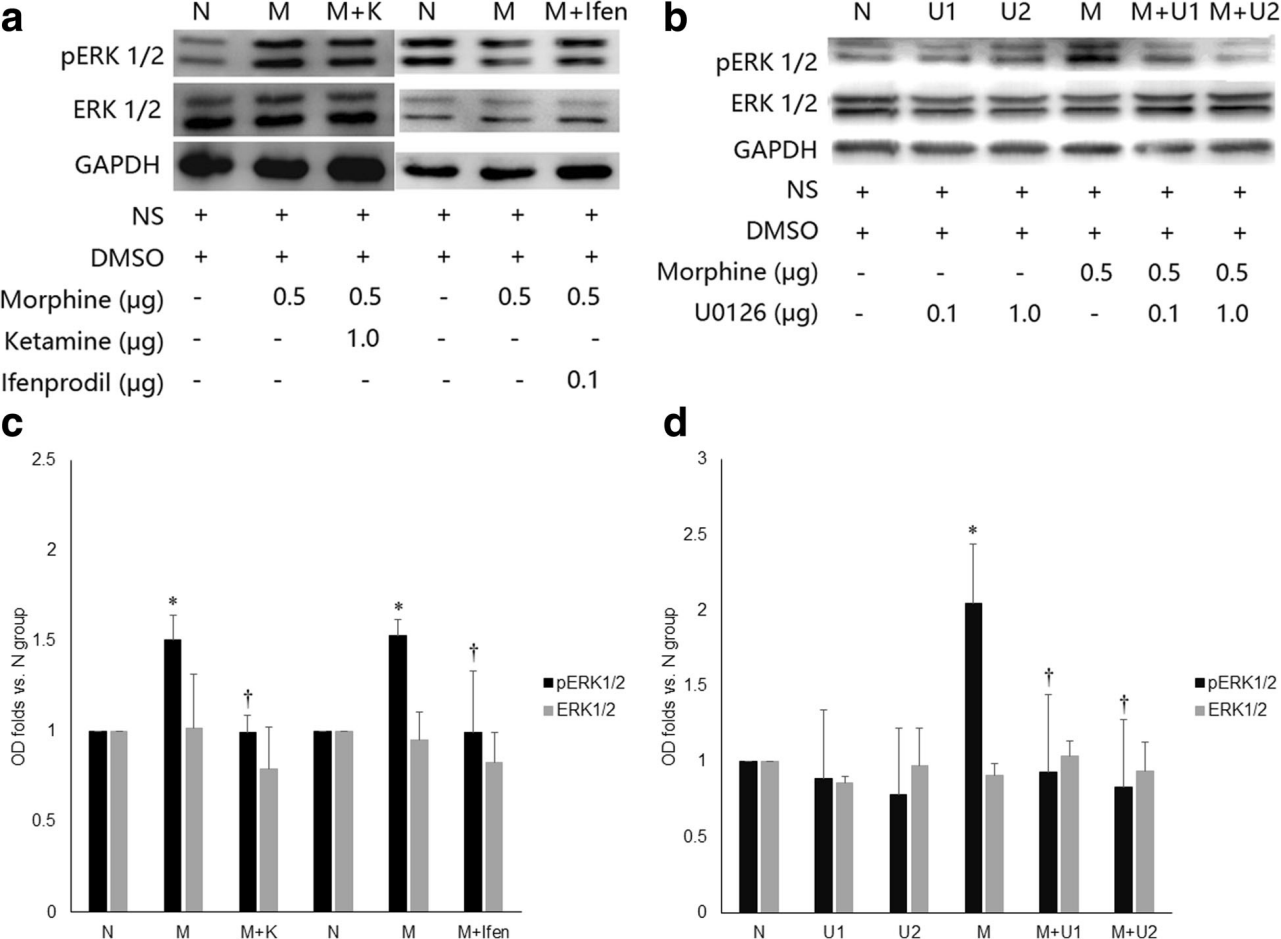
Expression and phosphorylation of ERK1/2 in the lumbar spinal cord dorsal horn. **a** & **c** The phosphorylation of ERK1/2 was increased in mice treated with 0.5 μg morphine (**m**) compared with that in the control group (**n**). Morphine-induced pERK1/2 expression was significantly decreased by 1 μg ketamine or 0.1 μg ifenprodil. **b** & **d** The phosphorylation of ERK1/2 was increased in mice treated with 0.5 μg morphine (**m**) compared with that in the control group (**n**) and the 0.1 μg (U1) and 1.0 μg (U2) U0126 groups. Morphine-induced pERK1/2 expression was significantly decreased by 0.1 μg (M+U1) or 1.0 μg (M+U2) U0126. There was no difference in the protein expression level of ERK1/2 among all groups. *: *p*<0.05, compared to the NS group, †: *p*<0.05, compared to the 0.5 μg morphine (M) group

Mice were sacrificed at 5 min post-injection, when the most obvious scratching behavior was exhibited. Compared with the NS group, the group given 0.5 μg morphine exhibited significantly greater phosphorylation of ERK1/2, which was inhibited by coadministration of 1.0 μg ketamine, 0.1 μg ifenprodil, or 0.1 μg or 1.0 μg U0126 (1.50±0.14 vs. 0.99±0.09, 1.53±0.09 vs. 0.99±0.34, 2.05±0.39 vs. 0.93±0.51, 2.05±0.39 vs. 0.83±0.45, respectively, *n*=6, *p*<0.05). In addition, there was no significant difference in the protein expression of ERK1/2 among any of the groups (Fig. 5).

### ERK1/2 phosphorylation inhibitor U0126 attenuated morphine-induced pruritus with no effect on its analgesia

**Fig. 6:**
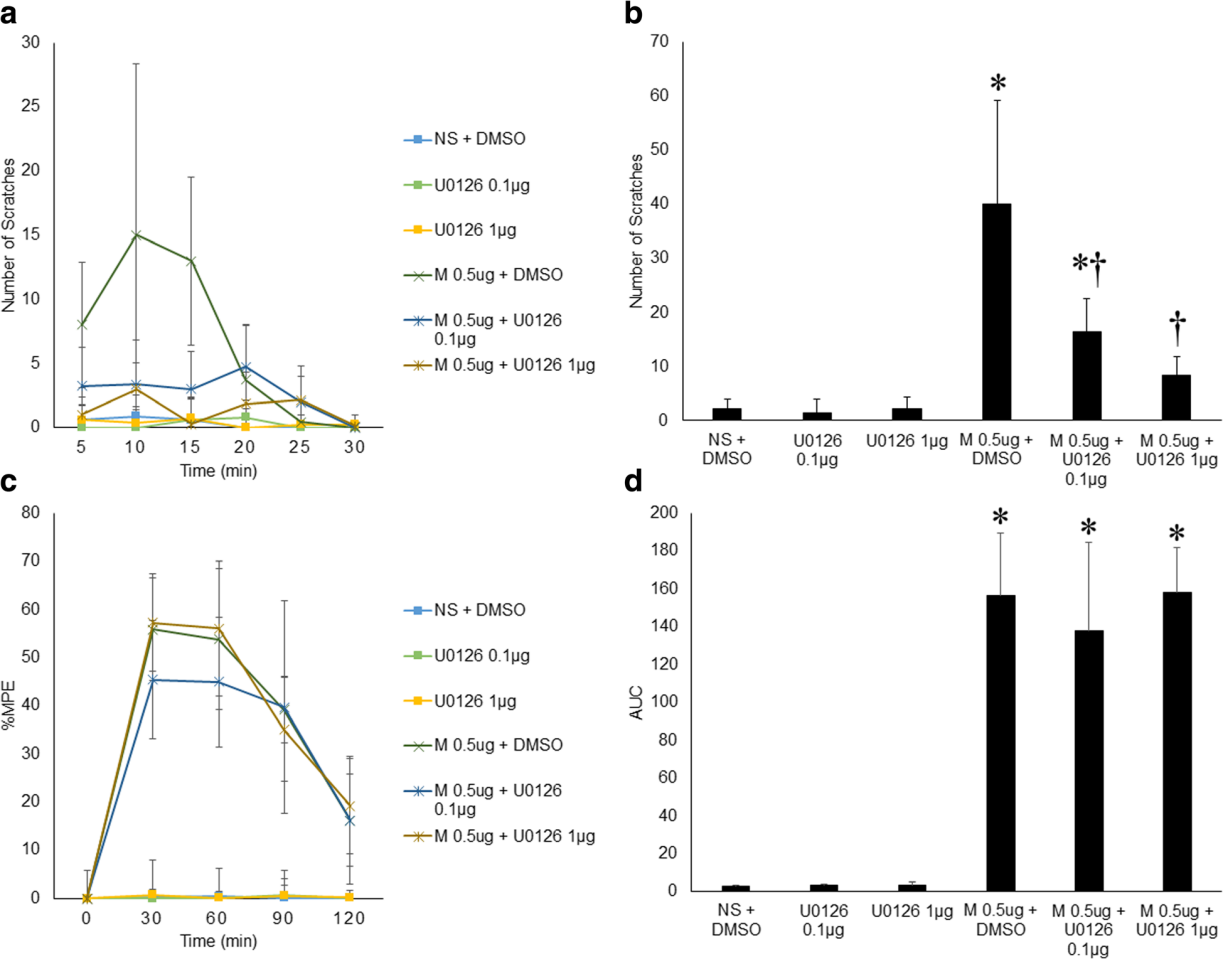
ERK1/2 phosphorylation inhibitor U0126 attenuated morphine-induced pruritus with no effect on its analgesia. **a** Morphine-induced pruritus (M 0.5 μg + DMSO) was obviously impaired by 0.1 μg or 1.0 μg U0126 in a dose-dependent manner. **b** U0126 (0.1 μg or 1.0 μg) significantly decreased the total number of scratches in 30 min that was induced by 0.5 μg morphine. **c** Time course of the effects of intrathecal morphine and intrathecal morphine + U0126 on morphine-induced analgesia during 2 h post-injection. %MPE showed no significant differences among the morphine group, morphine + 0.1 μg U0126 group and morphine + 1.0 μg U0126 group at 30, 60, 90 or 120 min post-injection. **d** The effect of U0126 on AUC calculated based on %MPE in (**c**). Compared with the AUC in the control group (NS + DMSO), the AUCs in the morphine group, morphine + 0.1 μg U0126 group and morphine + 1.0 μg U0126 group were all significantly increased. Error bars represent SEM. *: *p*<0.05, compared to the NS+DMSO group. †: *p*<0.05 compared to the 0.5 μg morphine + DMSO group

Both NMDAR antagonists, ketamine and ifenprodil, inhibited morphine-induced pruritus-related ERK1/2 phosphorylation, pruritus and analgesia. U0126, an ERK1/2 phosphorylation inhibitor, was tested at 0.1 μg and 1 μg in this study. Compared with the scratching response in the intrathecal 0.5 μg morphine group, the scratching response 30 min post-injection was significantly attenuated in the 0.5 μg morphine + 0.1 μg U0126 and 0.5 μg morphine + 1 μg U0126 groups (total number of scratching responses: 40.14±19.05 vs. 16.38±6.19, 40.14±19.05 vs. 8.43±3.31, respectively, *n*=9, *p*<0.05). Importantly, neither 0.1 μg nor 1 μg U0126 affected the time course of %MPE in those given 0.5 μg morphine (156.73±32.84 vs. 138.08±46.38, 156.73±32.84 vs. 158.04±23.83, respectively, *n*=9, *p*>0.05) (Fig. 6).

## Discussion

In this study, we demonstrated that intrathecal morphine injection elicited scratching responses in mice, which were reversed by NMDAR antagonists, ketamine and ifenprodil, in a dose-dependent manner. Additionally, coadministration of ifenprodil enhanced the analgesic effect of intrathecal morphine. Finally, the morphine-induced increase in phosphorylated ERK1/2 (pERK1/2) expression in the lumbar spinal cord was counteracted by coadministration of NMDAR antagonists.

### Morphine-induced scratching was different from morphine-induced analgesia

Morphine-induced scratching behavior and analgesia have been previously demonstrated to be mediated by two different subtypes of MOR, MOR1 and MOR1D, respectively [4]. The first part of our study revealed that, as the dose of intrathecal morphine increased from 0.1 μg to 0.25 μg, the scratching response increased slightly, and intrathecal injection of 0.5 μg morphine elicited significant scratching behavior. However, as the dose of morphine was further increased to 1.0 μg, the scratching responses were reduced, and some side effects of morphine, including fewer movements, respiratory depression, and movement instability, were observed. A time-course analysis revealed that the scratching responses peaked at 5 min and 15 min in all four morphine groups. In contrast, the time course of the morphine-induced analgesic response differed from that of the pruritic behavior. The analgesic efficacy of intrathecal morphine in mice reached its maximum at 30 min after injection and lasted for 2 h. When the morphine dose increased from 0.1 μg to 1.0 μg, the analgesic effect was stably enhanced. We further verified that the morphine-induced scratching behavior occurred independent of morphine-induced analgesia [4].

### NMDAR: a new therapeutic target of intrathecal morphine-induced pruritus

NMDAR is widely distributed throughout the central and peripheral nervous system and participates in the sensory processing and transmission of pain and itch, as well as in synaptic plasticity and central sensitization of neuropathic and inflammatory pain [14]. In neuropathic pruritus secondary to herpes zoster, which is unresponsive to standard medical therapy, modest control of the pruritus was achieved by a topical 0.5% ketamine gel [15]. In a rat model of pruritus induced by peripheral administration of 5-methoxytryptamine (MeOT), intrathecal administration of NMDAR antagonists decreased pruritic behavioral responses, and combinations of naltrexone with ketamine produced a prolonged antipruritic effect [16], suggesting an antipruritic function of NMDAR antagonists and a synergistic effect with MOR antagonists.

The second part of our study showed that coadministration of either NMDAR antagonist, ketamine (1 μg) or ifenprodil (0.1 μg), with 0.5 μg morphine completely abolished the scratching behavior compared with DMSO or no coadministered drug. Previous studies have proven that intrathecal injection of 3 μg ketamine or 0.5 μg ifenprodil or less does not impair motor function in mice [17]. For the first time, our findings demonstrated that NMDARs in the central nervous system are involved in intrathecal morphine-induced pruritus and that its antagonists could be used as potential therapeutics.

Currently the most effective treatment of intrathecal morphine-induced pruritus is an MOR antagonist, such as naloxone. However, the inability of an MOR antagonist to discriminate different subtypes of MORs might attenuate the analgesic effect of intrathecal morphine [2]. Considering the participation of the NMDAR in pain nociception and the well-known analgesic effect of ketamine [10, 18], we hypothesized that NMDAR antagonists co-injected with morphine could enhance the analgesic efficacy of morphine in mice. Surprisingly, we found that intrathecal coadministration of 1 μg ketamine and morphine did not affect morphine-induced analgesia. In clinical practice, the current evidence is insufficient to assess the benefits and harms of ketamine as an adjuvant to opioids for the relief of cancer pain, and the benefit of the addition of ketamine to patient-controlled epidural anesthesia (PECA) for postoperative pain management is also undefined [21, 19]. In contrast, coadministration of the NR2B selective NMDAR antagonist, ifenprodil, with morphine increased the analgesic efficacy of morphine in a dose-dependent manner. The affinities of ifenprodil to some other receptors and ion channel systems have been shown to be similar to its affinity to NMDARs [20], which might contribute to the antinociceptive effect and synergy with other analgesics [21, 22]. Consequently, the mechanisms of ifenprodil-induced analgesia by coadministration with morphine require further investigation.

Although ketamine neither impaired the analgesic efficacy of intrathecal morphine nor exhibited any side effects, its use in the central nervous system is limited by its neurotoxicity and is not recommend clinically [23]. The expression of the NR2B subunit is restricted to the spinal cord dorsal horn, thus resulting in fewer side effects and a wider therapeutic window than with the non-selective antagonist ketamine [20]. Therefore, ifenprodil could be a promising therapeutic option for intrathecal morphine-induced pruritus and optimization of postoperative intrathecal analgesia.

### The involvement of pERK1/2 in morphine-induced scratching responses and the therapeutic effects of NMDAR antagonists

ERK is one of the three major mitogen-activated protein kinases (MAPKs), including ERK1 and ERK2, which are often activated together and are collectively termed ERK1/2. ERK is activated, i.e., phosphorylated, by persistent neural activity and pathological stimuli [24]. Accumulating evidence indicates that pERK is a dynamic marker for neuron activation in the spinal cord [25]. Furthermore, activation of ERK signaling in the spinal cord is required for itch sensation in histamine and compound 48/80-treated mice [26]. Upon this study, we concluded that phosphorylation of ERK may contribute to intrathecal morphine-induced pruritus for following reasons: 1) We found that compared with the control group, mice treated with 0.5 μg intrathecal morphine exhibited elevated expression of pERK1/2 in the lumbar spinal cord dorsal horn at 5 min post-injection, which was in accordance with the peak of the scratching response. 2) In comparison with morphine alone, ketamine and ifenprodil co-injected with 0.5 μg morphine significantly decreased the phosphorylation of ERK1/2 and alleviated the scratching responses. 3) ERK1/2 phosphorylation inhibitor U0126 attenuated morphine-induced pruritus, but had no effect on its analgesia. Last but not least, morphine itself can induce the histamine release [27], as indicated that intrathecal morphine-induced pruritus might also be histamine-dependent.

Although substantial progress has been made in research on the itch sensation, pruritus is still a troublesome side effect of intrathecal morphine that lacks a consensus on the treatment and prevention strategy. In this study, we found that NMDAR antagonists ketamine and ifenprodil alleviated the scratching behavior induced by intrathecal morphine in mice. Furthermore, intrathecal coadministration of ifenprodil with morphine enhanced the analgesic efficacy of morphine alone. We also found that activation of ERK1/2 was related to intrathecal morphine-induced scratching and was counteracted by NMDAR antagonists.
